# UNDR ROVER - a fast and accurate variant caller for targeted DNA sequencing

**DOI:** 10.1186/s12859-016-1014-9

**Published:** 2016-04-16

**Authors:** Daniel J. Park, Roger Li, Edmund Lau, Peter Georgeson, Tú Nguyen-Dumont, Bernard J. Pope

**Affiliations:** Genetic Epidemiology Laboratory, School of Biomedical Sciences, Medical Building, The University of Melbourne, Melbourne, Victoria 3010 Australia; Department of Computing and Information Systems, The University of Melbourne, Melbourne, Victoria 3010 Australia; Victorian Life Sciences Computation Initiative, The University of Melbourne, Melbourne, Victoria 3053 Australia

**Keywords:** PCR-MPS, Hi-Plex, ROVER, Targeted sequencing, Massively parallel sequencing, Variant calling

## Abstract

**Background:**

Previously, we described ROVER, a DNA variant caller which identifies genetic variants from PCR-targeted massively parallel sequencing (MPS) datasets generated by the Hi-Plex protocol. ROVER permits stringent filtering of sequencing chemistry-induced errors by requiring reported variants to appear in both reads of overlapping pairs above certain thresholds of occurrence. ROVER was developed in tandem with Hi-Plex and has been used successfully to screen for genetic mutations in the breast cancer predisposition gene *PALB2*.

ROVER is applied to MPS data in BAM format and, therefore, relies on sequence reads being mapped to a reference genome. In this paper, we describe an improvement to ROVER, called UNDR ROVER (Unmapped primer-Directed ROVER), which accepts MPS data in FASTQ format, avoiding the need for a computationally expensive mapping stage. It does so by taking advantage of the location-specific nature of PCR-targeted MPS data.

**Results:**

The UNDR ROVER algorithm achieves the same stringent variant calling as its predecessor with a significant runtime performance improvement. In one indicative sequencing experiment, UNDR ROVER (in its fastest mode) required 8-fold less sequential computation time than the ROVER pipeline and 13-fold less sequential computation time than a variant calling pipeline based on the popular GATK tool.

UNDR ROVER is implemented in Python and runs on all popular POSIX-like operating systems (Linux, OS X). It requires as input a tab-delimited format file containing primer sequence information, a FASTA format file containing the reference genome sequence, and paired FASTQ files containing sequence reads. Primer sequences at the 5′ end of reads associate read-pairs with their targeted amplicon and, thus, their expected corresponding coordinates in the reference genome. The primer-intervening sequence of each read is compared against the reference sequence from the same location and variants are identified using the same algorithm as ROVER. Specifically, for a variant to be ‘called’ it must appear at the same location in both of the overlapping reads above user-defined thresholds of minimum number of reads and proportion of reads.

**Conclusions:**

UNDR ROVER provides the same rapid and accurate genetic variant calling as its predecessor with greatly reduced computational costs.

## Background

In recent work, we developed a highly multiplexed PCR-based target-enrichment system called Hi-Plex (www.hiplex.org) for massively parallel sequencing (MPS) [1]. Hi-Plex is a simple, low-cost protocol that can achieve highly accurate results. One of its key features is the ability to define a uniform library size which facilitates the removal of off-target amplification by size selection and, in combination with paired-end sequencing, allows complete overlap of read-pairs for each amplicon. The latter aspect permits a high degree of stringency in both the detection of variants and the filtering of artefacts caused by sequencing errors. Previously, we developed ROVER, a variant calling tool which takes advantage of the overlapping reads produced by Hi-Plex [2], and successfully applied Hi-Plex and ROVER to screening for genetic variants in the coding regions of *PALB2*, detecting all 60 variants identified by previous mutation screening and producing no false positive calls [3, 4].

ROVER requires as inputs a file describing the genomic coordinates of target amplicon regions in tab-delimited format and one or more sequence files in BAM format [5] containing paired-end reads mapped to a reference genome. It produces a list of variants in VCF format [6]. ROVER can detect single nucleotide variants (SNVs) and small insertions and deletions (indels). A variant is only reported by ROVER when it appears at the same position in both of the reads in an overlapping pair.

By far the most computationally expensive part of detecting variants with ROVER is the time taken to map (or align) the reads to the reference genome. In one indicative experiment, described below, the time taken for mapping with Bowtie (http://bowtie-bio.sourceforge.net/) [7] constituted approximately 78 % of the whole ROVER variant calling pipeline. Read mapping is a standard part of whole-exome and whole-genome DNA sequencing pipelines but, as has been demonstrated previously by Amplivar [8] and as we demonstrate in this paper in the context of Hi-Plex, it can be avoided in PCR-based MPS approaches. This is because the 5′ end of each read begins with a primer sequence whose genomic coordinates are already known. This latter information is determined during primer design. The reads do not need to be mapped to the reference because the primer-pairs identify the genomic coordinates of the intervening sequence. Optionally, we can further increase our confidence that a read is mapped to the correct location by checking that at least one of the reads in a pair is identical to the reference for a small sequence following the primer.

Having determined the starting coordinates of a particular read, we can then compare its sequence to the reference. To allow for insertions and deletions, it is necessary to perform a gapped alignment in the style of the Needleman-Wunsch algorithm [9]. However, the complexity of this algorithm is a quadratic function of the length of the aligned sequence and, therefore, expensive to compute for every read in the input. Fortunately, we can often avoid this cost because most reads in the input will be identical to the reference or will only differ by a small number of mismatches. In these cases, a simple linear comparison of the read to the reference is sufficient. We fall back to the gapped alignment algorithm only when the linear comparison fails.

Amplivar is based on premises similar to those underlying UNDR ROVER but applies a different mechanism. Amplivar uses primer sequences to associate reads with amplicons and reduces computational overheads by aligning reads as groups (using BLAT [10]). Furthermore, Amplivar merges overlapping reads (using SeqPrep [11]), whereas UNDR ROVER keeps both reads to test their concordance as part of a stringent filtering system.

UNDR ROVER was designed to support Hi-Plex targeted sequencing but is also compatible with other amplicon-based targeted sequencing systems that retain gene-specific primer sequences in the sequencing reads and for which primer and insert coordinates and primer sequences and paired FASTQ files can be supplied in the formats outlined in our documentation. AmpliSeq-generated data would not be compatible, for example, because the gene-specific primers are largely cleaved during library generation. A key consideration is that UNDR-ROVER is intended to work with sequencing data exhibiting considerable overlap of read-pairs - as such, it is not recommended for use with systems that do not achieve this.

## Implementation

UNDR ROVER is implemented in Python 2.7 as a command-line application. Its four mandatory arguments are: 1) a tab-delimited format file which associates primer-pairs with their genomic coordinates; 2) a tab-delimited format file which matches primer names to their insert sequences; 3) a FASTA format file containing the reference sequence (the primers must have been designed from this reference to ensure that the coordinates agree); 4) one or more pairs of FASTQ files. The main output of UNDR ROVER is a VCF file containing the detected variants. Additionally, it produces two log files which report on the overall execution of the program and the depth to which each amplicon is covered by the reads.

**Fig. 1:**
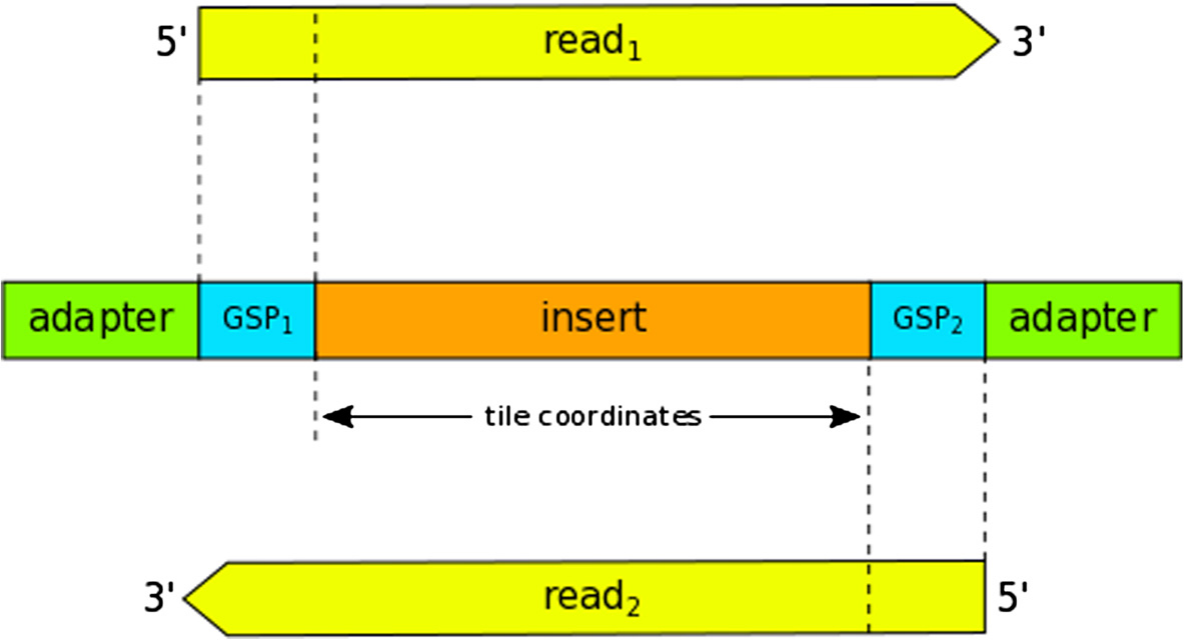
Hi-Plex library structure and overlapping reads. The center rectangle represents the target insert DNA sequence flanked by gene-specific primer (GSP) sites (blue) and adapter sequences (green). The two reads of a pair are shown in yellow. The 5′ end of each read starts with its corresponding gene-specific primer sequence. The insert size is chosen so that both reads overlap the target insert sequence completely. The 3′ ends of reads may extend into the adapter sequence depending on the read length and the presence/absence of insertions/deletions in the template DNA. The diagram is not to scale. Typically, the insert sequence will be significantly longer than the primer sequences

Hi-Plex employs PCR to amplify selected target regions of DNA. Larger segments of DNA are split into tiles of a specified narrow size range (typically, the order of 100 nucleotides). The regular tile size facilitates size selection of the amplified product which increases on-target stringency and allows both reads of a pair to overlap the entire tile. Hi-Plex is compatible with short-read sequencing platforms such as Illumina TruSeq (MiSeq and HiSeq instruments, Illumina, San Diego, CA, USA) and Ion Torrent (PGM and Proton instruments, Life Technologies, Carlsbad, CA, USA) [12]. Hi-Plex primers consist of a pool of relatively low concentration (individually) gene-specific primers (GSPs) that seed the PCR, and universal adapter primers that drive the majority of the reaction. GSPs are designed to correspond to the sequences flanking the target inserts. Figure 1 illustrates the structure of a Hi-Plex library element in relation to the two overlapping reads of a read-pair.

UNDR ROVER comprises two main steps: 1) associating reads with their corresponding primer tiles and 2) calling variants.

A primer tile is a contiguous section of the genome which is flanked by a primer-pair. We take advantage of the fact that the 5′ end of each read starts with a GSP sequence for which the coordinates are known. Therefore, the start of each read can be compared to the full set of GSPs to identify its corresponding tile. This comparison is made efficient by storing primer information in a hash table indexed by primer sequence. As such, the coordinates of each read can be found in time proportional to the length of the primer sequence. Hi-Plex primers can vary in length to a small degree, therefore UNDR ROVER stores only the first N bases of primers in the hash table. The value of N is user definable and should be no larger than the length of the shortest primer used in the experiment. This scheme requires that the first N bases of a read is an exact match to its corresponding primer sequence and, therefore, does not tolerate mismatches derived from errors in the sequencing chemistry and/or production of primer oligonucleotides. Reads which do not start with a known primer sequence are discarded. We have not found this to be a problem in practice due to the high fidelity of modern MPS platforms, especially at the 5′ end of reads. In the example experiment described below, out of a total of over 13 million reads, 84 % matched exactly with a primer sequence.

UNDR ROVER uses the same variant calling algorithm as its predecessor, ROVER, which requires that both reads in a pair overlap their associated tile by at least a specified percentage (by default 90% of the tile must be overlapped by each read). Reads which do not satisfy this requirement are discarded. Some provision to allow incomplete overlap is engineered to accommodate contexts that preclude the achievement of complete ‘tiling’, such as the presence of genomic insertion events or intractable sequences for primer design. For additional stringency, UNDR ROVER can optionally test whether, in at least one of the reads in a pair, the sequence just after the primer sequence is an exact match with the corresponding target reference sequence. By default, this test will use a sequence of 30 nucleotides, but it can be configured by a command-line argument. Since only one read of a read-pair (at either end) is required to match the expected sequence for a read-pair to contribute to variant calling, variants that are present in the terminal regions are detectable unless they coincide with additional variants at the other end of the read-pair.

UNDR ROVER compares the expected insert sequence from the reference genome to the part of the read following the GSP. In the common case we expect the sequences to be identical or only have one base mismatch. Therefore, as an optimisation, UNDR ROVER first performs a linear comparison of the two. If more than one mismatch is detected by the linear comparison, then a gapped-alignment of the two sequences is performed using the Needleman-Wunsch algorithm as implemented in the *pairwise2* module of BioPython [13]. In the majority of cases the linear comparison is sufficient, thus avoiding the significantly greater cost of gapped alignment most of the time. Additional speed can be achieved (with the possibility of slightly increasing the error rate) optionally by allowing more than one mismatch to occur in the linear comparison if each mismatch is separated by less than or equal to a specified number of bases. We use the term *thorough* when no more than one mismatch in the linear comparison is permitted, and the term *fast* for the more lenient option.

As with its predecessor, UNDR ROVER only calls variants which appear in both reads of a pair such that 1) the frequency of the variant-pair is above a minimum absolute value, 2) the variant-pair occurs above a minimum percentage of all read-pairs overlapping the amplicon, and 3) each read of a pair overlaps the target amplicon by a user-defined minimum percentage. All of these conditions can be adjusted by command line arguments. Optionally, UNDR ROVER can filter out any bases which do not meet a minimum base-quality score.

**Fig. 2:**
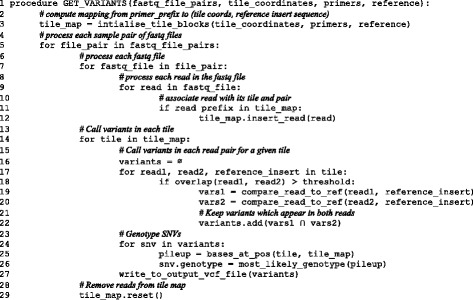
Pseudo code for variant calling algorithm employed by UNDR ROVER

We illustrate UNDR ROVER’s algorithm with the pseudo-code in Fig. 2. The algorithm is realised by the GET_VARIANTS procedure which takes four parameters: 1) a sequence of paired-end FASTQ files, one pair for each input sample; 2) the list of tile coordinates associated with primer names; 3) a list of primer names associated with corresponding DNA sequences; 4) a reference genome sequence. The output is a VCF file containing variants and associated metadata such as frequency count, genotype and whether they passed various filtering tests.

A hash table mapping primers to their corresponding tiles is intialised (line 3). The keys of the hash table are length N prefixes of the primer DNA sequences. The values associated with each key contain the genomic coordinates of the tile plus the reference sequence at the same location. Primer pairs are connected by having the entry for the reverse primer point back to the entry for its forward partner. Each pair of FASTQ files is processed in sequence (lines 5 to 29); UNDR ROVER calls variants in each input sample separately. For each sample, all reads in each FASTQ file are associated with their corresponding tile (lines 6 to 12), then variants are called for each tile (lines 13 to 28). Each read is associated with its tile by hashing the first N bases of the sequence and looking up the result in the tile map. Each read in a pair is compared to the corresponding reference sequence and differences between the two are computed (lines 19 and 20) using the approach described earlier. Differences which appear on both read pairs are retained (line 21). Frequencies for each variant are computed and the filters described above are applied. Each SNV is genotyped by computing a pileup of bases at the position of the variant. The most likely genotype is computed by comparing the expected distribution of DNA bases for a given pileup coverage size to the actual distribution of bases. The expected distribution is computed from the ploidy of the putative genotype and a very simple error model, which assumes a constant read error rate which defaults to 1/500, but can be overridden as a command line parameter. By default, UNDR ROVER assumes a diploid genotype model, but this can be overridden to a haploid model via a command line argument. The distance between the expected and actual base frequency distributions are computed using a statistical G-Test which is based on a log-likelihood ratio. The genotype with the smallest distance to the observed data is taken to be best explanation for the observed data. Genotyping can add extra time to variant calling and is therefore only performed when an optional command line argument is set.

## Results

We have demonstrated the performance of UNDR ROVER by comparing it to its predecessor ROVER using a previously published dataset [3]. Hi-Plex was used to screen 95 blood-derived DNA samples targeting the protein coding and some flanking intronic and untranslated regions of *PALB2* and *XRCC2* using 60 primer-pairs in the PCR. The resulting library was sequenced on a MiSeq instrument (Illumina) producing 95 pairs of FASTQ files (190 files in total) with an average file size of 23 MiB. Previous application of ROVER to this dataset (aligned to the entire human genome (hg19) using bowtie2-2.1.0) accurately detected all 60 variant occurrences identified through mutation screening and assigned no false positive calls. Application of UNDR ROVER to the same data set yielded the same set of called variants in both thorough and fast modes. Future experiments will seek to validate UNDR ROVER using additional data sets that, similar to the data set used in this study, have been extensively characterised by Sanger sequencing.

**Fig. 3:**
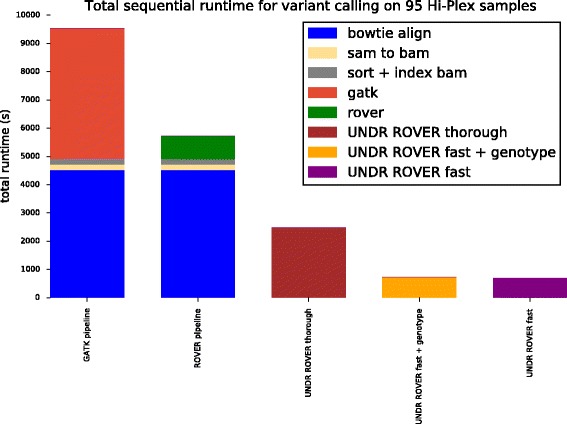
Runtime comparison of GATK, ROVER and UNDR ROVER. Total sequential computing time of the GATK pipeline, ROVER and UNDR ROVER (thorough, genotyping and fast) when applied to 95 Hi-Plex samples targeting *PALB2* and *XRCC2* with 60 primer-pairs in the PCR. The computing time for the GATK and ROVER pipelines are decomposed into alignment with Bowtie (blue), conversion of alignment file from SAM to BAM format (yellow), indexing and sorting of BAM file (grey), and variant calling (light red for GATK and green for ROVER). Computing times for UNDR ROVER are shown for both the thorough mode (brown) and the fast mode with SNV genotyping (orange), and the fast mode without SNV genotyping (purple)

We also applied the GATK HaplotypeCaller (version 3.4-46) [14] variant calling software to the same samples, after aligning the FASTQ files to the reference with Bowtie. We instructed GATK to only call variants in the targeted regions. GATK called the same variants as ROVER and UNDR ROVER plus two additional calls which appear to be false positives caused by sequencing artefacts. Both ROVER and UNDR ROVER were able to filter out these false positives because the artefacts did not appear on both reads in the affected read pairs. The GATK command used to call variants is shown below:
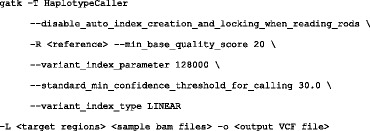


Figure 3 compares the total sequential computing time for UNDR ROVER against the GATK and ROVER pipelines (including read alignment with Bowtie) when applied to the entire set of 95 samples. We applied UNDR ROVER in three different modes to show the performance implications of different settings. The performance tests were executed on a single node of an IBM iDataPlex cluster with 16 core 2.7GHz CPUs and 256GB of RAM using the Red Hat Enterprise Linux version 6 operating system. Total sequential computation times for computing variants in all 95 Hi-Plex input samples were 9535 s for the GATK-based pipeline, 5721 s for the ROVER-based pipeline, 2480 s for UNDR ROVER in thorough mode without genotyping, 736 for UNDR ROVER in fast mode with genotyping, and 690 s for UNDR ROVER in fast mode without genotyping. Approximately 78 % of the ROVER pipeline time is constituted by read alignment with Bowtie. This highlights the significant performance gains possible by avoiding the alignment step. In summary, for this indicative experiment, we see that UNDR ROVER is able to achieve between a 2-fold and 8-fold performance improvement compared to its predecessor, and up to 13-fold improvement over a pipeline based on GATK, whilst producing the same set of variant calls.

## Discussion

The following command line illustrates a typical invocation of UNDR ROVER:
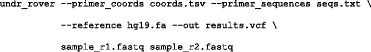


where the input files coords.tsv and seqs.txt provide the primer coordinates and DNA sequences, respectively, hg19.fa is the reference DNA sequence and sample1_r1.fastq and sample_r2.fastq contain the input DNA reads. The output variant calls are written to the file results.vcf.

Below is a short example primer coordinates file illustrating two pairs of primers:
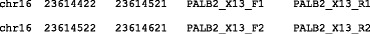


The first column indicates the chromosome of the targeted sequence. The second and third columns indicate the start and end coordinates of the target tile. The fourth and fifth columns indicate the unique symbolic names of the forward and reverse primers for a target tile.

Below is a short example primer sequences file, with entries corresponding to the gene-specific portions of primers from the example coordinates file above:
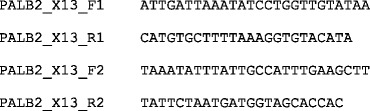


The first column indicates the unique symbolic name of the primer and the second column indicates the primer DNA sequence written in the 5′ to 3′ direction. Primer coordinates are matched with primer sequences via their unique symbolic names.

## Conclusions

UNDR ROVER provides a computationally more efficient alternative to ROVER and other standard variant calling pipelines for the detection of genetic variants from Hi-Plex-generated datasets while maintaining a high level of accuracy.

## Availability and requirements

Project name: UNDR ROVER

Project home page: https://github.com/bjpop/undr_rover

Usage instructions: http://bjpop.github.io/undr_rover

Operating systems: POSIX-like operating systems (OS X, Linux)

Programming language: Python

Other requirements: PyVCF, Pyfaidx, BioPython and SciPy libraries

## Abbreviations

GSP: gene specific primer
MPS: massively parallel sequencing
SNV: single nucleotide variant
VCF: variant call format
